# Adipose, Bone Marrow and Synovial Joint-Derived Mesenchymal Stem Cells for Cartilage Repair

**DOI:** 10.3389/fgene.2016.00213

**Published:** 2016-12-20

**Authors:** Christopher R. Fellows, Csaba Matta, Roza Zakany, Ilyas M. Khan, Ali Mobasheri

**Affiliations:** ^1^Faculty of Health and Medical Sciences, University of SurreyGuildford, UK; ^2^Department of Anatomy, Histology and Embryology, Faculty of Medicine, University of DebrecenDebrecen, Hungary; ^3^Centre for NanoHealth, Swansea University Medical SchoolSwansea, UK; ^4^Arthritis Research UK Centre for Sport, Exercise and Osteoarthritis, Queen's Medical CentreNottingham, UK; ^5^King Fahd Medical Research Center, King AbdulAziz UniversityJeddah, Saudi Arabia; ^6^Sheik Salem Bin Mahfouz Scientific Chair for Treatment of Osteoarthritis with Stem Cells, King AbdulAziz UniversityJeddah, Saudi Arabia

**Keywords:** Mesenchymal stem cell (MSC), articular cartilage, adipose tissue, bone marrow, synovial joint, tissue engineering

## Abstract

Current cell-based repair strategies have proven unsuccessful for treating cartilage defects and osteoarthritic lesions, consequently advances in innovative therapeutics are required and mesenchymal stem cell-based (MSC) therapies are an expanding area of investigation. MSCs are capable of differentiating into multiple cell lineages and exerting paracrine effects. Due to their easy isolation, expansion, and low immunogenicity, MSCs are an attractive option for regenerative medicine for joint repair. Recent studies have identified several MSC tissue reservoirs including in adipose tissue, bone marrow, cartilage, periosteum, and muscle. MSCs isolated from these discrete tissue niches exhibit distinct biological activities, and have enhanced regenerative potentials for different tissue types. Each MSC type has advantages and disadvantages for cartilage repair and their use in a clinical setting is a balance between expediency and effectiveness. In this review we explore the challenges associated with cartilage repair and regeneration using MSC-based cell therapies and provide an overview of phenotype, biological activities, and functional properties for each MSC population. This paper also specifically explores the therapeutic potential of each type of MSC, particularly focusing on which cells are capable of producing stratified hyaline-like articular cartilage regeneration. Finally we highlight areas for future investigation. Given that patients present with a variety of problems it is unlikely that cartilage regeneration will be a simple “one size fits all,” but more likely an array of solutions that need to be applied systematically to achieve regeneration of a biomechanically competent repair tissue.

## Introduction

Osteoarthritis (OA) is the progressive loss of normal joint function and is characterized by the degeneration of articular cartilage leading to pain, stiffness, and loss of joint mobility. OA is the world's leading cause of physical disability in adults, in the UK 20% of adults aged between 50–59 and 50% aged between 80–89 have symptomatic disease in one or both knee joints (Lacey et al., 2008). Current treatments for OA generally involve managing pain and inflammation, physiotherapy and lifestyle modifications in order to slow the progression of disease. When conservative forms of therapy are exhausted, patients and their rheumatologists may elect to have surgery.

Joint cartilage degeneration has multiple causes; biomechanical stress, metabolic and genetic defects, and, in many cases first manifests as a focal chondral or osteochondral lesion. Lesions can spread over the rest of the joint surface as the edges of the defect are more susceptible to degenerative change due to inequalities in biomechanical stress (Lefkoe et al., 1993; Braman et al., 2005). Much research has focused on repairing focal surface defects in order to spare the joint from further degeneration and recover pain free movement. A fundamental barrier to restoration of cartilage integrity is its slow intrinsic repair capacity that is largely attributable to a lack of vascularity, relatively low cellularity in adult tissue, and the presence of a dense hydrated extracellular matrix that inhibits cellular migration to and from the site of injury. Repair of lesions above a critical size of 3–5 mm in diameter must be initiated through extrinsic mechanisms such as microfracture of the subchondral bone plate to release blood and marrow into the defect to form a clot and fibrocartilaginous repair tissue (Pridie, 1959). A related technique, autologous chondrocyte implantation (ACI), is a two-part procedure, first the isolation and expansion of chondrocytes from intact non-weight-bearing regions of the joint and secondly their transplantation into an osteochondral defect in a weight-bearing region (Grande et al., 1989; Brittberg et al., 1994). The limited expansion capacity of dedifferentiated chondrocytes and their increasing inefficiency at redifferentiation during extended culture has led to the use of mesenchymal stem cells (MSCs) for autologous cartilage repair of larger defects (Wakitani et al., 1994). MSCs are found in numerous human tissues including bone marrow and adipose tissue. These MSCs have been shown to differentiate into bone, cartilage, muscle, and adipose tissue. Further studies have also shown cells displaying the properties of adult-derived MSCs can be isolated from the synovial joint tissues such as articular cartilage and synovial membrane (Alsalameh et al., 2004; Dowthwaite et al., 2004; Williams et al., 2010; McCarthy et al., 2012).

Cell-based therapy is becoming an established element of modern healthcare and is predicted to grow as knowledge and implementation of cell biology, biomaterials, and regenerative medicine increases. The aim of this article is to provide an overview of mesenchymal stem cell-based therapies for the treatment of lesion in articular cartilage. In the absence of disease modifying pharmacological agents (Mobasheri, 2013a) or biological therapies (Mobasheri, 2013b), cell-based therapies are one of the few available treatments for focal cartilage and osteoarthritic defects. In this review article we describe the significance of this topic in the context of the biology of the joint and the osteoarthritic disease process, and summarize key concepts and developments in the area of MSC-based therapies.

## Articular cartilage and the chondrocyte

**Figure 1:**
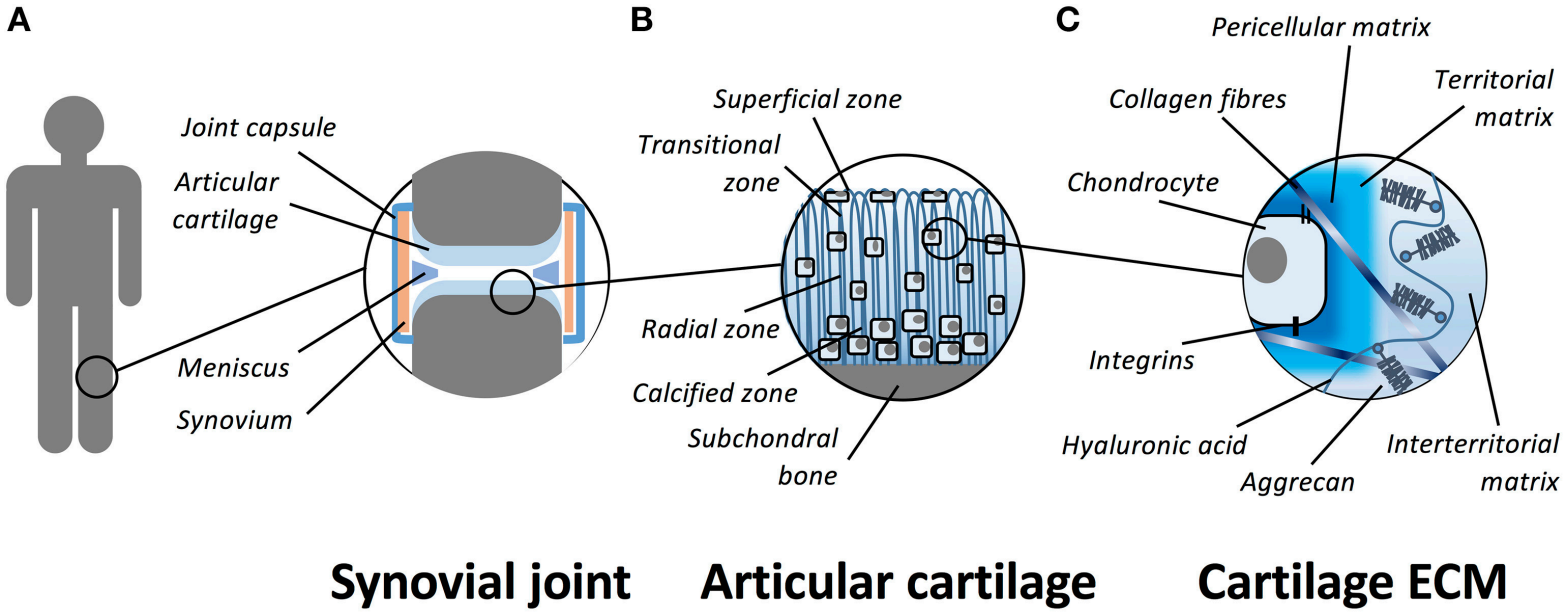
**Schematic representation of the organization of articular cartilage**. **(A)** Articular cartilage is a thin layer of hyaline tissue that covers the surface of bones in synovial joints. **(B)** The tissue is divided into four zones; the superficial (tangential), transitional, radial, and calcified zones. **(C)** The hyaline cartilage matrix can be subdivided by its composition around chondrocytes as pericellular, territorial and inter-territorial matrices. The collagens (primarily collagen type II, IX, and XI) provide tensile strength and the main proteoglycan, aggrecan, is responsible for compressive stiffness. Chondrocyte-matrix interactions are mediated through plasma membrane receptors such as integrins and CD44.

Articular cartilage is a smooth, thin and opalescent layer of hyaline tissue that covers the surface of bones of synovial joints (Figure 1). It is a highly organized and specialized tissue that permits free articulation, painless movement, and transmission of force through the skeleton due to the avascular, alymphatic and aneural composition. Compared to other tissues, articular cartilage has a low rate of metabolic activity (Pearle et al., 2005). The tissue is maintained by a single sparsely distributed cell, the chondrocyte (Archer and Francis-West, 2003a), that is surrounded by a highly hydrated extracellular matrix (ECM). The structure of cartilage is primarily composed of a highly organized collagen network that supports aggregating proteoglycans (PGs) and hyaluronan (HA) that is the most abundant non-PG forming glycosaminoglycan (GAG) in the cartilage matrix (Jeffery et al., 1991). Articular cartilage is divided into four zones; the superficial (tangential), transitional, radial, and calcified zones (Eyre, 2002). The physical and biochemical differences between the zones is important to allow cartilage to resist both extrinsic and intrinsic forces (Knudson and Knudson, 2001). In addition to the pseudo-stratified appearance of the matrix, it can be further subdivided by its composition around chondrocytes as pericellular, territorial and inter-territorial matrices. The collagen fibril arrangement and orientation, cellular density and proteoglycan composition vary with depth to provide the diverse mechanical properties required across the joint. The main collagens (type II, IX, and XI) provide tensile strength and the main proteoglycan, aggrecan, delivers compressive stiffness. Small proteoglycans, including decorin, biglycan, and fibromodulin, bind to other matrix macromolecules and thereby help to stabilize the matrix (Buckwalter and Mankin, 1998). Chondrocyte-matrix interactions are mediated through the pericellular and territorial matrices, facilitated by collagen type VI and non-collagenous proteins such as anchorin CII, tenascin, and fibronectin (Poole et al., 2001). This allows mechanical, electrical, and physicochemical signal transduction to the chondrocytes, directing the synthetic and degradative activity of chondrocytes (Mobasheri et al., 2002; Millward-Sadler and Salter, 2004). Cartilage tissue contains a large proportion of water (65–80% by wet weight). Chondrocytes themselves constitute 5–10% of the tissue's total volume, collagens form 10–30%, whilst proteoglycans and other molecules consist of 5–10% of the tissue's wet weight (Eyre, 2002; Archer et al., 2003b; Hunziker et al., 2007).

In adult cartilage, the prime function of articular chondrocytes is to synthesize and maintain the ECM required for tissue function. As the tissue is not vascularized it relies on diffusion from the articular surface for nutrient and metabolite exchange; therefore, chondrocytes are adapted to operate at a low oxygen tensions (ranging from 5% at the surface to <1% in the mid/deep layers) with the majority of the cell's energy requirements coming from glycolysis (Lafont, 2010). Despite mature articular chondrocytes having a relatively low metabolic rate, they display a remarkable synthetic capacity. The cartilage ECM is continually remodeled as chondrocytes replace matrix macromolecules lost through degradation as part of normal turnover (Goldring and Marcu, 2009). ECM maintenance depends on the ability of chondrocytes to detect alterations in the macromolecular composition and organization of the matrix, such as the presence of degraded macromolecules, and to respond by synthesizing appropriate types and amounts of new ECM components. The balance between catabolism and anabolism of the ECM maintains the healthy cartilage structure; a loss of equilibrium can lead to degeneration (Goldring and Marcu, 2009; Bader et al., 2011). During aging and disease, tissue homeostasis is disrupted; the rate of loss of ECM exceeds the rate of deposition of newly synthesized molecules (Goldring and Marcu, 2009). Although articular cartilage can tolerate repetitive physical loading, it has a low capacity for self-repair, therefore, an inability to quickly heal minor injuries makes cartilage particularly sensitive to progressive damage and degenerative diseases such as OA.

## Cartilage damage and OA

Articular cartilage defects caused by trauma or chronic injury are classified into two categories: Partial-thickness and full-thickness (Hunziker, 1999). Partial-thickness or chondral defects do not penetrate to the subchondral bone and therefore rely on healing through intrinsic processes that are limited by the tissue's inherent characteristics. There is evidence of transient chondrocyte proliferation at wound sites, however, there is little or no cellular migration into the injured sites to repair the defect (Hunziker and Rosenberg, 1996; Zhang et al., 2009). Furthermore, the reparative response usually terminates before the cartilage defect is fully healed, resulting in a lasting defect of mainly acellular tissue that reduces tissue function leading to further tissue degeneration (Hunziker, 1999; Zhang et al., 2009).

Full-thickness osteochondral defects penetrate the entire thickness of articular cartilage to the subchondral bone. Unlike partial-thickness defects, full-thickness defects are accessible to MSCs, macrophages, and blood cells, if there is a break in the underlying subchondral bone plate. In the latter case, a fibrin clot fills the defect and MSCs from bone marrow migrate into the repair matrix. Gradually MSCs replace the fibrin clot, stem cells differentiate into chondrocytes and secrete a proteoglycan-rich ECM to remodel the damaged area (Hunziker, 1999). However, it is frequently reported that a fibrous repair tissue is formed, which has weaker biomechanical properties and higher permeability (Kreuz et al., 2006). Consequently, the spontaneous repair process in full-thickness defects is a transient phenomenon and tissue degeneration eventually re-occurs. At this point, complete resurfacing is rarely observed leading to the development of secondary OA.

OA is a disease that affects the whole joint, including cartilage, subchondral bone, synovium, tendons, and muscles (Sellam and Berenbaum, 2010; Goldring, 2012; Loeser et al., 2012; Berenbaum et al., 2013; Mobasheri et al., 2014). The disease is characterized by degeneration of articular cartilage, low-grade synovial inflammation, and alterations in the joint soft tissues and subchondral bone (Goldring and Goldring, 2007; Sellam and Berenbaum, 2010; Mobasheri et al., 2014). Most commonly, OA occurs as primary or idiopathic OA; risk factors include obesity, overuse, joint instability, genetic or anatomical irregularities, metabolic disorders, muscle weakness, and various disorders of bone turnover (Mobasheri et al., 2014). The disease progresses in three stages; firstly there are changes in or loss of the ECM, then chondrocytes attempt to remodel and repair the tissue and finally, the degeneration occurs to a greater extent than synthesis causing incremental loss of cartilage (Hendren and Beeson, 2009). Cartilage degeneration results in bone-to-bone articulation, inflammation, significant pain, and disability. A major component of OA is inflammation, there is an increased activity of many cytokines, chemokines, and adipokines (Loeser et al., 2012). The inflamed synovium produces catabolic and pro-inflammatory mediators such as cytokines, nitric oxide, prostaglandin E_2_ (PGE_2_), and neuropeptides. These molecules unbalance the matrix degradation and repair equilibrium resulting in excessive secretion of the proteolytic enzymes and cartilage ECM breakdown (Goldring and Goldring, 2007; Sellam and Berenbaum, 2010). Cartilage alterations induce further synovial inflammation, creating a progressive loop that exacerbates clinical symptoms and stimulates further joint degradation.

As cartilage ages, there are a number of biochemical and morphological changes that occur within the tissue that alter the mechanical properties and decrease the ability of chondrocytes to maintain articular cartilage (Martin and Buckwalter, 2002). Damage-induced cellular alterations result in a senescence-associated secretory phenotype characterized by the production and secretion of cytokines, chemokines, and proteases (Loeser, 2013; Mobasheri et al., 2014). Oxidative stress and alterations in mechanical loading can further promote changes in the chondrocytes. OA is an active, progressive, degenerative disease that is the common pathway of age related damage, acute injury and inflammatory degradation of synovial joints.

## Cartilage regeneration and repair

As mentioned previously cartilage has a poor intrinsic repair capacity, tears and defects larger than 3 mm rarely heal and potentially can lead to OA of the joint. Poor repair is partly due to the lack of blood vessels, which are required for efficient repair responses, and, because of a low cell density with chondrocytes embedded within lacunae, unable to easily migrate to damaged areas (Henrotin et al., 2005). Therefore, extrinsic intervention is often necessary to repair damaged tissue and to prevent further damage. The most effective treatment for end-stage OA is arthroplastic prosthetic replacement. However, due to the finite lifespan of prostheses, this is unsuitable for patients younger than 45 years. A variety of treatment methods have been used to repair defects and stimulate the formation of new articular cartilage. These include lavage, microfracture, osteochondral allograft transfer system (OATS), ACI and osteotomy (Bhosale and Richardson, 2008). Each procedure has varied success and many factors have to be considered including defect size and patient age when selecting the correct treatment.

The most widely used method that surgeons adopt for cartilage repair involves penetration of the subchondral bone causing the release of multipotent MSCs in a process defined as marrow stimulation. Previous iterations of this method have included Pridie drilling, spongialization and abrasion therapy, though the most popular modern method is microfracture (Ronn et al., 2011). Under arthroscopy, tiny fractures are made using an awl in the subchondral bone, inducing bleeding and a fibrin clot that is infiltrated with stem cells from the bone marrow. Stem cells fill the defect, differentiate and form a repair tissue (Yen et al., 2008) that is fibrocartilaginous and not suitable to withstand the demands of everyday activities, and thus, at higher risk of future breakdown (Redman et al., 2005). Microfracture, however, does offer symptomatic relief; in a follow up study it was reported that pain was decreased and function improved in 95% of the study population after up to 17 years post-surgery (Steadman et al., 2003).

An alternative intervention developed by Brittberg and colleagues, ACI, is currently regarded as the “gold standard” for cartilage repair (Brittberg et al., 1994). Cartilage is harvested from a low weight-bearing region, chondrocytes are isolated and expanded *in vitro* before being injected into a full-thickness articular defect under a periosteal patch stitched over the defect and sealed in with fibrin glue (Brittberg et al., 1994, 2003; Redman et al., 2005). Implanted chondrocytes begin the process of producing neo-cartilage through the production of ECM. ACI has been shown to produce effective and durable repair tissue, relieving symptoms and clinical success remains high, even after 20 years post-implantation (Peterson et al., 2010). The repair tissue produced by ACI has been shown to be varied but in general is more hyaline-like than produced using microfracture. However, there is often an abundance of type I collagen which is also characteristic of fibrocartilage (Roberts et al., 2002). Improvements in the procedure have led to second generation ACI techniques; synthetic collagen membranes have replaced the periosteal flap, and several biomaterial and natural scaffolds have been developed into which the chondrocytes are seeded (Redman et al., 2005). Despite the encouraging clinical outcomes ACI has a number of disadvantages; it requires multiple surgeries and is more invasive than microfracture, treatable defect size is limited by the finite amount of harvestable donor tissue and the restricted *in vitro* expansion of chondrocytes before de-differentiation makes their use redundant (Barbero et al., 2003). In follow-up studies, it has been shown that 1 year post-operatively, ACI offers significantly improved repair compared to microfracture (Visña et al., 2004); however, after 2–5 years randomized trials show no significant difference in repair efficiency between ACI and microfracture (Knutsen et al., 2007; Van Assche et al., 2010). The limitations of current surgical strategies have led to investigations into the use of adult stem cells from various tissue sources in an endeavor to improve hyaline-like cartilaginous repair and increase the treatable defect size.

## MSC physiology, and function

Friedenstein first characterized clonogenic fibroblast-like cells extracted from bone marrow *via* attachment to tissue culture plastic (Friedenstein et al., 1976). These marrow-derived stromal cells were found to be inherently osteogenic but displayed plasticity being capable of differentiating into multiple cell types of the mesodermal lineage. MSCs have been shown to form cartilage, bone, adipose tissue, intervertebral disc, ligaments, and muscle (Prockop, 1997; Pittenger et al., 1999). Therefore, MSCs are typically defined as adherent, self-renewing, fibroblastoid-like cells that can differentiate to osteoblasts, adipocytes, and chondrocytes *in vitro* (Barry and Murphy, 2004; Phinney and Prockop, 2007). Self-renewal refers to the biological pathways and mechanisms that preserve the undifferentiated stem cell state. In MSCs this capacity for self-renewal is in part due to telomerase reverse transcriptase (TERT) activity (Kolf et al., 2007). Additionally, leukemia inhibitory factor (LIF), fibroblast growth factors (FGFs), Wnts and other growth factors and cytokines, have been implicated in maintenance of the MSC phenotype (Tsutsumi et al., 2001; Metcalf, 2003; Kléber and Sommer, 2004; Kolf et al., 2007). These factors have also been shown to be critical for self-renewal and maintenance of undifferentiated embryonic mesenchymal tissue.

It is widely accepted that primary MSC cultures are a heterogeneous population of cells with varying capacities of self-renewal and differentiation (Ho et al., 2008; Phinney, 2012). Their heterogeneity means no singular unique marker is available for identification and isolation (Table 1). Therefore, a panel of positive and negative markers must be used for the selection criteria. MSC populations commonly express surface proteins including CD29, CD44, CD49a–f, CD51, CD73, CD90, CD105, CD106, CD166, and Stro1 and must be negative for hematopoietic lineage markers including CD11b, CD14, and CD45 (Halfon et al., 2011). The optimal panel of marker for selection is frequently debated and numerous additional markers have been reported in the literature. The International Society of Cellular Therapy recommended that MSCs must meet a minimum criteria including: Adhesion to plastic, expression of surface markers CD73, CD90, CD105 (≥95% expression), the absence of hematopoietic markers CD34, CD45, CD14 or CD11b, CD79α or CD19 (≤2%), the absence of HLA Class II molecules and tripotent differentiation into chondrogenic, osteogenic, and adipogenic phenotypes (Horwitz et al., 2005; Dominici et al., 2006).

**Table 1:** **Cell Surface markers for undifferentiated MSCs**.

**Tissue reservoir**	**Positive markers**	**Negative markers**	**References**
Bone marrow	CD13, CD29, CD44, CD49a–f, CD51, CD73, CD90, CD105, CD106, CD166, and STRO-1	CD11b, CD14, CD19 CD34, CD45, and CD79α	Halfon et al., 2011; Barry and Murphy, 2013
Adipose	CD44, CD90, and CD105	CD45, CD34, and CD133	Dominici et al., 2006
Cartilage	CD9, CD49e, CD90, CD166, Notch1, and STRO-1	CD45, CD133/−1, and −2	Alsalameh et al., 2004; Fickert et al., 2004; Williams et al., 2010
Synovial membrane	CD44, CD90, CD105, and CD147	CD34, CD45, CD117, and CD31	De Bari et al., 2001b; Sakaguchi et al., 2005
Synovial fluid	CD10, CD13, CD40, CD44, CD55, CD73, CD90, CD 105, CD 166, and D7-FIB	CD11b, CD34, CD45, and CD271	Jones et al., 2004; Krawetz et al., 2012
Fat pad	CD13, CD29, CD44, CD90, and CD105	CD34, CD56, CD271, and STRO1	Khan et al., 2012
Periosteal membrane	CD9, CD73, CD90, CD105, SH2, SH3, and SH4	CD11b, CD14, CD19, CD34, CD45, CD79a, and HLA-DR	Lim et al., 2005; Chang and Knothe Tate, 2012
Trabecular bone	CD44, CD64, CD90, CD105, CD147, and CD166	CD31, CD34, CD45, and CD117	Sakaguchi et al., 2004
Muscle	CD34, CD144, and CD56		Zheng et al., 2007

**Figure 2:**
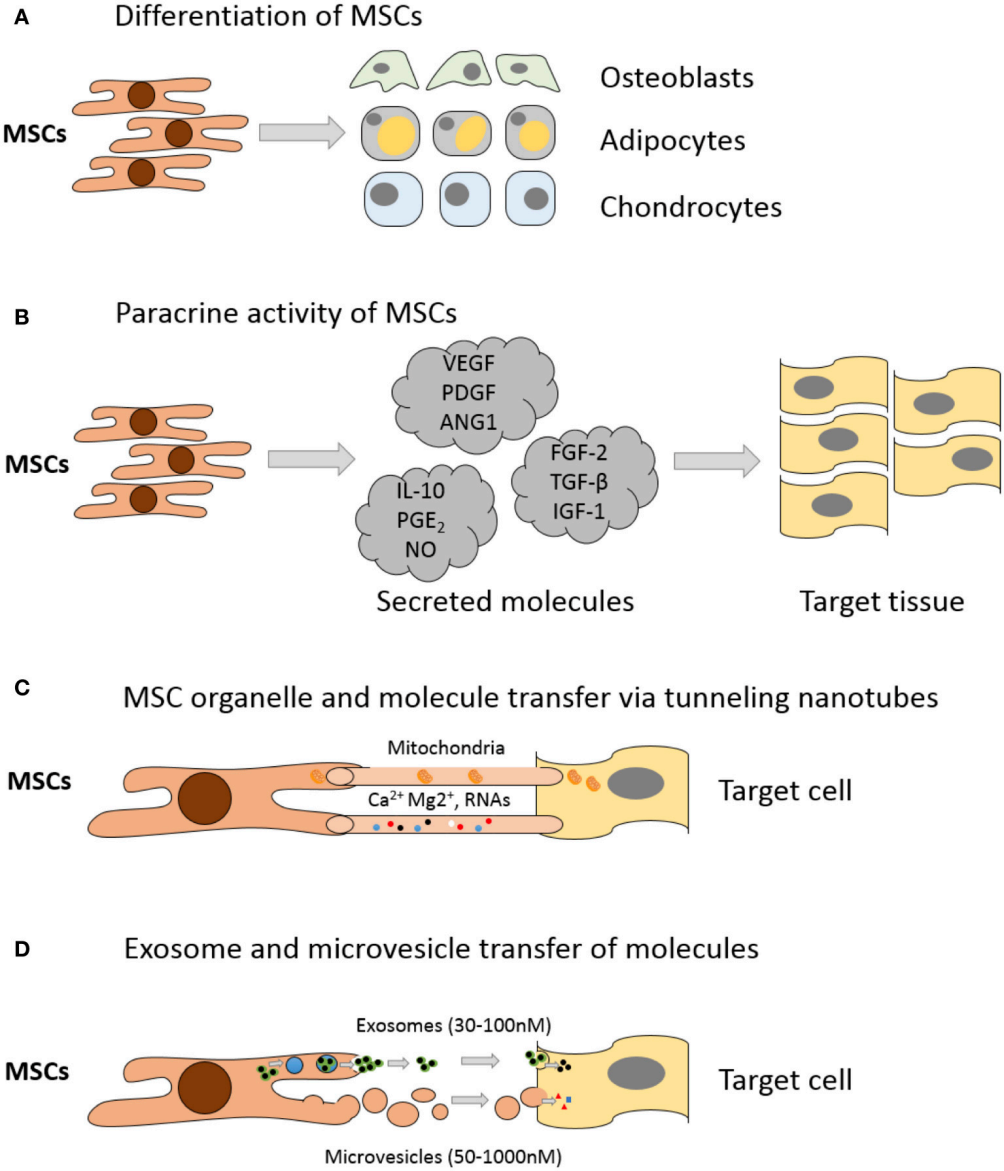
**Mechanisms of MSC mediated repair**. **(A)** Differentiation into replacement cell types. **(B)** Secretion of paracrine factors such as growth factors, cytokines, and hormones. Vascular endothelial growth factor (VEGF), platelet-derived growth factor (PDGF), angiopoietin-1 (ANG1), interleukin-10 (IL-10), prostaglandin E_2_ (PGE_2_), nitrous oxide (NO), fibroblast growth factor (FGF-2), Transforming growth factor-beta (TGF-β), insulin-like growth factor-1 (IGF-1). **(C)** Transfer of organelles (e.g., mitochondria) and/or molecules through tunneling nanotubes. **(D)** Transfer of proteins/peptides, RNA, hormones, and/or chemicals by extracellular vesicles such as exosomes or microvesicles. Exosomes are generated through the endocytic pathway and released through exocytosis. Microvesicles are produced by cell surface budding and released directly from the plasma membrane. Adapted from Spees et al. (2016).

Whilst MSC capacity for multipotent differentiation represents a great potential for regenerative medicine, beneficial effects are also achieved by MSC-mediated improvements in native cell viability and proliferation, reduction in apoptosis and anti-inflammatory immunomodulatory effects (Caplan, 2009; Glenn and Whartenby, 2014). These additional reparative effects are achieved through MSC secretion of paracrine growth factors and cytokines, direct cell-cell interactions through tunneling nanotubes and release of extracellular vesicles containing peptides, mRNA and microRNAs (Figure 2); for a detailed review of the various MSC repair mechanisms please see Spees et al. (2016). Treatment of articular cartilage with intra-articular injection of MSCs has shown that some regeneration of joint tissues can occur; however, studies have shown that the source of repair tissue is primarily derived from native cells (Murphy et al., 2003). In support of the latter studies, Horie and colleagues showed that inter-articular injection of human MSCs into the rat knee increased the expression of type II collagen in resident cells (Horie et al., 2012). These findings indicate that MSCs also coordinate and enhance the reparative response rather than exclusively replacing lost and injured tissue (Wyles et al., 2015). MSC-enhanced repair is induced via secretion of various anabolic paracrine factors, such as transforming growth factor beta (TGF-β), vascular endothelial growth factor (VEGF), fibroblast growth factor (FGF) and other bioactive molecules that modulate reparative responses (Tille and Pepper, 2002; Sorrell et al., 2009; Freitag et al., 2016). Chondrocytes from end stage diseased cartilage remain metabolically active and continue to express genes for cartilaginous matrix molecules, corroborating the hypothesis that MSCs support repair and modulate these chondrocytes (Freitag et al., 2016).

Recent studies have revealed that MSCs interact with immune cells and eliminate survival factors, suppress inflammatory T cell proliferation, inhibit maturation of monocytes and myeloid dendritic cells, and re-calibrate the chemokine gradient (Glenn and Whartenby, 2014). MSCs inhibit several aspects of B cell activity, including activation, proliferation, chemokine receptor expression, and differentiation into antibody-secreting plasma cells (Glenn and Whartenby, 2014). MSCs have also been shown to induce nitric oxide (NO) in response to inflammatory cytokines to suppress CD8+ T cell proliferation, cytokine production, and cytotoxicity (Glenn and Whartenby, 2014). MSCs also produce many soluble factors including TGF-β, hepatocyte growth factor (HGF), PGE_2_ and interleukin 10 (IL-10) that suppress CD4+ T cell proliferation and polarize them toward T-helper cells (TH1/TH2) and anti-inflammatory T-regulatory cells (Tregs) (Glenn and Whartenby, 2014), promoting T cell apoptosis and reduce inflammation, and providing a favorable environment for tissue repair. After *in vivo* administration, MSCs can induce peripheral tolerance and migrate to injured tissues where they have the capacity to exert immunosuppressive properties (Glenn and Whartenby, 2014) and inhibit the release of pro-inflammatory cytokines and promote the survival of existing cells and the repair of damaged tissue (Uccelli et al., 2008). Using a murine *in vivo* model of OA it has been shown that MSCs deactivate macrophages stimulated by cartilage breakdown products, preventing further catabolism of the cartilage and also elevating macrophage production of anabolic growth factors (ter Huurne et al., 2012). Due to their strong immunomodulatory phenotype MSCs are being explored clinically for treatment of a variety of immune-mediated diseases such as motor neuron disease and Sjögren's syndrome (Parekkadan and Milwid, 2010).

The versatility of MSCs has led to the development of cellular therapies for tissue engineering and regenerative medicine. According to data reported by the US National Institutes of Health (http://www.clinicaltrial.gov/) there are approximately 500 clinical trials currently utilizing MSCs as therapy for a diverse range of disease (Squillaro et al., 2016). MSCs seem to provide clear benefits over chondrocytes when treating degenerative conditions such as OA. MSCs can be expanded for a prolonged period *ex vivo* due to their capability of self-renewal and also maintain their phenotype to a greater degree than dedifferentiated chondrocytes in culture. Additionally, MSCs have the potential to form all joint tissues and this hypothetically enables them to repair both lesions in the articular cartilage or osteochondral defects as well as other joint structures such as ligaments.

## Tissue reservoirs of MSCs

**Figure 3:**
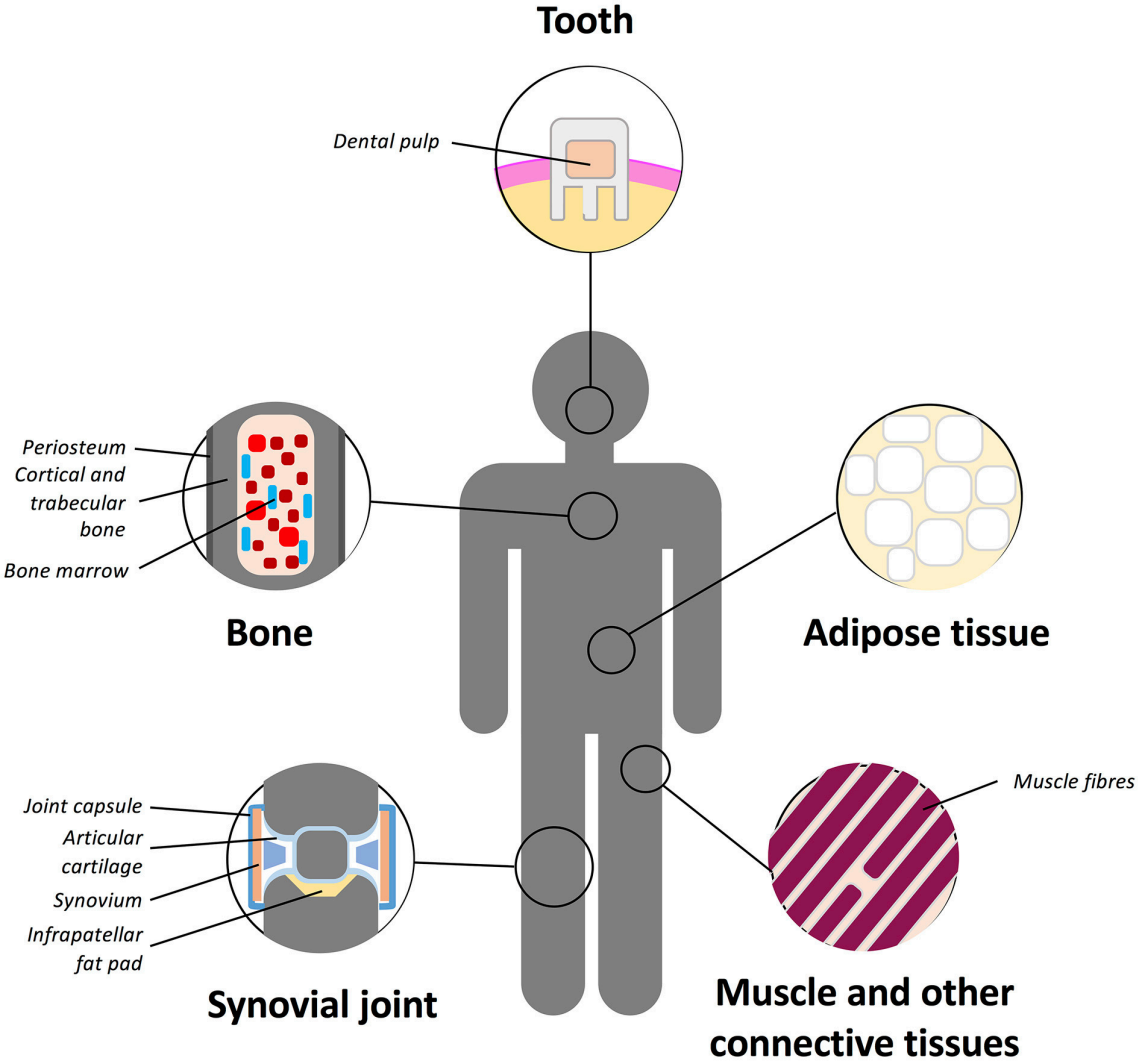
**Schematic representation of the main tissue reservoirs of MSCs in the human body**. MSCs have been found in the bone marrow, adipose tissue, articular cartilage, synovium, skeletal muscle, dental pulp, circulatory system, heart, brain, umbilical cord (including Wharton's jelly), and other connective tissues. Refer to text for further details.

The “niche” incorporates all the factors required to regulate stem cell self-renewal and differentiation including differentiated or stromal cells in direct or indirect contact with stem cells, ECM molecules and soluble mediators within the milieu (Schofield, 1978; Kolf et al., 2007). It is believed that certain signals enter the niche or that stem cell leave the niche in order to initiate differentiation for regeneration or repopulation of a tissue (Kolf et al., 2007). Multiple MSC niches have been described within various tissues (Figure 3) and stem cells housed in them isolated and utilized for chondrogenic repair. MSCs have been found in the bone marrow, adipose tissue, articular cartilage, synovium, skeletal muscle, dental pulp, circulatory system, heart, brain, umbilical cord tissues (including Wharton's jelly), and other connective tissues (Gronthos et al., 2000; Zuk et al., 2001; Alsalameh et al., 2004; Dowthwaite et al., 2004; Crisan et al., 2008; Gronthos and Zannettino, 2011; Batsali et al., 2013; Mobasheri et al., 2014; Steward and Kelly, 2015).

### Bone marrow

Bone marrow is currently the most common MSC source in the clinical practice and have been extensively used for cartilage repair either alone or with biological scaffolds. However, there are some limitations with bone marrow-derived MSCs (BM-MSCs), in that they are a rare population occurring with a frequency of 0.01–0.001% (Bonab et al., 2006). In addition, the differentiation potential of BM-MSCs decreases with increased expansion *in vitro* (Banfi et al., 2000; Li et al., 2011) and harvesting these cells involves a painful procedure and causes donor site morbidity.

A clinical study of BM-MSC transplantation for cartilage repair using passaged bone marrow MSCs suspended in a collagen type I gel and transplanted with an autologous periosteal flap showed that 42 weeks post-surgery hyaline cartilage-like tissue was partially present. That study did not find any significant clinical improvement above the controls; however, the arthroscopic and histological grading scores were better in the cell-transplanted cohort (Wakitani et al., 2002). The same authors also reported patients treated with BM-MSC-containing scaffolds, secured with a periosteal or synovial flap, had fibro-cartilaginous repair tissue 12 months post-surgery (Wakitani et al., 2007). Nejadnik and colleagues performed an observational cohort study in which the clinical outcomes of patients treated with ACI were compared with outcomes of patients treated with autologous bone marrow MSCs (Nejadnik et al., 2010). Using BM-MSCs in cartilage repair was found to be at least as effective as chondrocytes for articular cartilage repair in improving symptoms of patients. BM-MSC repair strategies also had the additional advantage of requiring one less knee surgery and reducing costs. However, multiple studies have reported that chondrogenically induced BM-MSCs hold the inherent risk of either forming transient fibrocartilagenous repair tissue or undergoing terminal differentiation to form calcifying cartilage, subchondral bone overgrowth or intralesional osteophytes (Shapiro et al., 1993; McCarthy et al., 2012). For these reasons other types of MSCs are being actively researched.

### Adipose

In the last two decades, numerous publications proved the multipotent nature of an undifferentiated cell population, which can be obtained from adipose tissue or liposuction samples and which are now classified as adipose derived stem cells (ASCs) (Zuk et al., 2001; Gimble and Guilak, 2003). These cells have been shown to exhibit the potential for osteogenesis, chondrogenesis, adipogenesis, myogenesis, as well as certain levels of neurogenesis in numerous studies (Erickson et al., 2002; Adams et al., 2012; Feng et al., 2014; Deshpande et al., 2015; Mellor et al., 2015). Despite the fact that ASCs show similarities to bone marrow MSCs, they exhibit a number of distinct characteristics, for example in their cell surface markers, differentiation potential, and abundance in the body. The great advantage of ASCs is in their yield which when compared to 100 ml of bone marrow aspirate, up to 300-fold more stem cells can be obtained from 100 g of adipose tissue (Aust et al., 2004; Oedayrajsingh-Varma et al., 2006).

Another advantage of ASCs application is the relative ease of the isolation of this cell population. Isolation of MSCs from adipose tissue includes several very simple steps that can be performed by anybody with routine cell culture laboratory experience. If the sample is a native adipose tissue it has to be minced and washed extensively to remove as much blood as it is possible. In case of liposuction samples the first step is unnecessary. In order to liberate cells from the stroma of the adipose tissue, samples are incubated with collagenase Type I followed by a gentle centrifugation (around with 1200 g) of the digest. The floating population of mature adipocytes can be removed and the pelleted portion of the samples can be regarded as stromal vascular fraction (SVF). SVF contains various cell types, such as endothelial cells, fibroblasts, white blood cells, macrophages and the subject of our interest, pericytes/multipotent stromal cells. These multipotent mesenchymal cells can be selected from this mixed cell population on the basis that they are strongly adherent to plastic cell culture surfaces, thus the final step for the ASC isolation is the establishment of adherent cell cultures of SVF (Estes et al., 2010; Yu et al., 2011). These adherent cells have slim, elongated fibroblast/mesenchymal cell-like morphology that is preserved throughout the culturing process. If clinical application is the aim of the ASC separation, circumstances of the isolation and expansion procedures have to fulfill the requirements for Good Manufacturing Practice (cGMP) guidelines, which could cause significant delays in any clinical application of the obtained ASCs. Nonetheless, a routine cell culture lab is fully suitable for isolation and culturing of ASCs applied for basic research purposes. Recently, an innovative method and device named Lipogems® have been developed (Bianchi et al., 2013) with which the lipid content of lipoaspirates becomes significantly lower and a remarkably cell-rich lipid-derivative with intact blood vessels surrounded by high number of pericytes can be separated. The Lipogems® product is regarded as processed with minimal manipulation [Regulation (EC) No. 1394/2007 of the European Parliament and of the Council; http://eur-lex.europa.eu/]; therefore, direct autologous application of samples obtained in this way for clinical therapy, such as lipid replacing plastic surgery interventions or intraarticular injection with orthopedic indications, can be performed (Tremolada et al., 2016).

In order to be classified as MSCs isolated ASCs have to express cell surface markers accepted for identification of mesenchymal cells, such as CD44, CD90, and CD105, and are expected to lack expression of the haematopoietic stem cell markers CD45, CD34, CD133 (Dominici et al., 2006). However, ASCs also exhibit various other cell markers which are associated with stemness such as POU5F1, NANOG and KLF4 (Dudakovic et al., 2014; Lv et al., 2014; Camilleri et al., 2016). ASCs may exhibit individual variations of these marker patterns amongst patients or depending on the locations of the source-fat deposit (i.e., subcutaneous or visceral) in the body (Yang et al., 2014). A common observation of numerous investigators is that cryopreservation or long term culturing and expansion of ASCs, similarly to BM-MSCs, reduces their viability and differentiation potential (Gonzalez-Fernandez et al., 2015). In summary, the above facts clearly demonstrate that ASCs represent a multipotent cell population which is a very promising candidate for successful tissue engineering of cartilage, bone and other skeletal tissues.

Independent of the methods of isolation, ASCs have demonstrated potential for chondrogenic differentiation when expanded in appropriate monolayer conditions (Estes et al., 2008) and then cultured with chondrogenic factors in a 3D environment (Erickson et al., 2002). However, results are mixed with some studies reporting ASCs have inferior chondrogenic potential in comparison to BM-MSCs (Afizah et al., 2007), whilst others not finding any significant difference between the two cell types (Ronziere et al., 2010). Various growth factors have been shown to promote *in vitro* cartilage formation of ASCs, amongst them the effectiveness of TGF-β1, IGF-1 (Zhou et al., 2016), and BMP-6 (Hennig et al., 2007) has been well established. Besides the soluble chemical regulators of cartilage formation, a wide range of chondrogenesis promoting scaffolds have been developed and introduced. The scaffolds support 3D culture and aim to mimic the extracellular environment of chondrocytes in order to favor chondrogenic differentiation. Chitosan, collagen, alginate, or gelatine-based scaffolds, as well as hydrogels have been successfully applied for this purpose (Focaroli et al., 2014; Dinescu et al., 2015; Ogawa et al., 2015). High molecular weight HA alone or in combination with other scaffold materials has also been proven to enhance chondrogenesis of ASCs by several groups (Son et al., 2013; Wu et al., 2013). Recently, ASCs transduce with an inducible interleukin 1 receptor antagonist (IL-1Ra) transgene were seeded on the surface of an anatomically shaped poly-caprolactone scaffold. That study showed that advanced textile manufacture when combined with gene delivery into ASCs can produce large and shaped cartilaginous tissue that produces anti-inflammatory cytokines (Moutos et al., 2016).

External physical factors, particularly optimal mechanical load seem to play a significant positive role either during *in vivo* or *in vitro* formation of musculoskeletal tissues, including cartilage (for a recent review see Trumbull et al., 2016). However, only a few studies have been published about the role of mechanical forces in the chondrogenic differentiation of ASCs. Mechanical load reduced the type I collagen content of bio-fabricated cartilage generated from porcine bone marrow and infrapatellar fat pad (IFP) derived ASCs, and in case of marrow-derived MSCs, calcification was also lowered by the mechanical stimuli, but the efficacy of cartilage formation was not unambiguously enhanced (Luo et al., 2015). In other studies, porcine, rabbit or human ASCs seeded into various scaffolds and subjected to plated compression or cyclic hydrostatic pressure respond to mechanical stimuli with augmented chondrogenesis (Correia et al., 2012; Li et al., 2012; Carroll et al., 2014). Moreover, Carrol and colleagues proposed that responsiveness of multipotent ASCs to external physical cues might be considered as a functional assay to evaluate their therapeutic potential. Two recent reviews summarize efforts to fabricate or regenerate articular cartilage with application of ASCs (Wu et al., 2013; Bielli et al., 2016). Therefore, current evidence demonstrates that adipose-derived MSCs can provide a readily available cells source that have shown clinical promise for articular cartilage repair (Perdisa et al., 2015).

### Resident MSCs within the synovial joint tissues

#### Articular cartilage

Development of articular cartilage progresses through appositional growth driven by a progenitor/stem cell subpopulation that resides in the articular surface (Archer et al., 2003b). Adult articular cartilage was thought to be devoid of stem cells or progenitors but recent work by multiple groups has demonstrated their presence *in vivo* and *in vitro* (Alsalameh et al., 2004; Dowthwaite et al., 2004; Fickert et al., 2004; Grogan et al., 2009; Williams et al., 2010; Pretzel et al., 2011; Nelson et al., 2014). Tissue specific MSCs have now been isolated from healthy and osteoarthritic adult articular cartilage. These cells are capable of multipotent differentiation, preferentially bind fibronectin and exhibit a high colony forming efficiency, and they have been shown to grow for over 70 population doublings *in vitro* (Williams et al., 2010). Cartilage-derived chondroprogenitors (CPCs) are colony-forming cells enriched by differential adhesion to fibronectin and are positive for CD90, CD105, CD166, STRO-1, Notch-1 and negative for CD45 and CD34 cell surface markers and able to undergo multi-lineage differentiation. Bovine and human articular CPCs retain SOX9 expression and exhibit telomere maintenance after extensive cell expansion of clonal isolates, and retain their multipotent differentiation capacity (Khan et al., 2009; Williams et al., 2010).

The discovery of cartilage-derived progenitors, whilst a useful cell source for tissue engineering applications, has inevitably led to a greater focus on their role in intrinsic repair processes particularly during OA disease. Chondrocyte clusters are a well-defined feature of osteoarthritic cartilage and are hypothesized to result as a proliferative response to injury where cells are aberrantly constrained within a single lacuna due to unknown reasons (Lotz et al., 2010). The presence of a highly mitotic subpopulation of cells within osteoarthritic cartilage suggests that progenitor cells exist within the tissue either the result of chondrocyte dedifferentiation or re-activation.

In the absence of specific phenotypic markers for CPCs a number of studies have used fluorescence-activated cell sorting for various combinations of MSC cell surface antigens including CD9, CD44, CD90, and CD166 to detect progenitor-like subpopulations in osteoarthritic cartilage. Results show that 2–17% of cells within OA cartilage exhibit antibody reactivity to MSC-like cell surface determinants and can undergo tri-lineage differentiation to form fat, bone and cartilage (Alsalameh et al., 2004; Fickert et al., 2004; Pretzel et al., 2011). Colony-forming assays using differential adhesion to fibronectin and Hoescht dye exclusion assay have also been used to identify CPCs in osteoarthritic cartilage, where two studies found no difference in their frequency between normal and diseased cartilage (Grogan et al., 2009; Nelson et al., 2014). Koelling et al. were able to isolate a highly proliferative population of CPCs from human osteoarthritic cartilage solely through their enhanced migratory ability (Koelling et al., 2009). *In vitro* studies have shown hypocellular cartilage formed by blunt impact or scratching can be repopulated by adjacent chondroprogenitor-like cells with their defining characteristic being overexpression of markers for cellular proliferation (Seol et al., 2012). Studies using blunt force trauma to replicate aspects of post-traumatic OA have similarly identified chondrocytes capable of proliferation and Notch-1 expression as those capable of promoting an instrinsic repair response (Henson et al., 2005). Therefore, current evidence demonstrates that normal, injured and diseased articular cartilage contain a viable pool of progenitor cells with mesenchymal stem cell-like characteristics with an inherent potential for maintenance and repair.

There are clear advantages associated with the use of articular cartilage-derived MSCs in cell-based cartilage therapy. Firstly, these cells are isolated from local tissue and have been shown to possess sufficient proliferative capacity for expansion without losing their propensity for chondrogenic differentiation. Compared to MSCs from bone marrow and adipose-derived progenitors, articular cartilage-derived stem cells are believed to be further along in their commitment to the chondrogenic lineage, primed to differentiate to form hyaline cartilage, making them a logical choice for tissue engineering (Jayasuriya and Chen, 2015). Moreover, articular CPCs, unlike marrow stromal mesenchymal cells do not terminally differentiate toward an epiphyseal lineage producing hypertrophic chondrocytes. CPCs do not generate a collagen type X-rich matrix or express RUNX2 transcription factor protein but make predominantly collagen type II and a hyaline cartilage-like matrix upon chondrogenic differentiation (Williams et al., 2010; McCarthy et al., 2012). Stem cell populations within articular cartilage offer a potentially novel and promising cell source for tissue engineering. Identification of reliable markers and isolation methods are essential in ensuring the most effective repair.

#### Synovium and synovial fluid

MSCs derived from synovial membranes (SM-MSCs) can be expanded *in vitro* for prolonged periods with limited cell senescence and can be consistently induced into multilineage differentiation pathways regardless of donor age or serial passage (Nishimura et al., 1999; De Bari et al., 2001a). They express MSC phenotypic markers and are negative for hematopoietic markers as shown in Table 1. Unlike CPCs and BM-MSCs, SM-MSCs *in vitro* do not exhibit telomerase activity, it is therefore postulated that the telomere length and capacity for self-renewal is regulated through telomerase independent mechanisms (Blasco et al., 1999; De Bari et al., 2001a). Previous studies have shown that SM-MSCs have greater chondrogenic potential than donor matched BM-MSCs, ASC, and periosteum- or muscle-derived stem cells (Sakaguchi et al., 2005; Futami et al., 2012). When chondrogenic differentiation was induced with BMP-2, TGF-β and dexamethasone in 3D pellet culture, synovial-derived MSCs consistently produce larger cartilaginous pellets than bone marrow MSCs from the same patients (Shirasawa et al., 2006). Intra-articular injection of SM-MSCs in rat, rabbit, porcine and primate meniscal defect and injury models have shown that SM-MSCs can promote meniscus regeneration and provide protection of medial femoral articular cartilage (Hatsushika et al., 2013, 2014; Ozeki et al., 2015; Kondo et al., 2016). In early clinical trials SM-MSCs have shown promise for treatment of articular cartilage defects, producing hyaline cartilage repair tissue (Sekiya et al., 2015). In a 3-year follow-up study, defects transplanted with SM-MSCs showed improved MRI, qualitative histology and Lysholm scores (Sekiya et al., 2015). The advantage that synovial MSCs hold over other cells sources is they can be grown to first passage in only 14 days. Transplantation of synovial MSCs is also less invasive for the patient than OATS or ACI.

MSCs have also been found in synovial fluid (SF-MSCs) of the normal knee joint and their number increases over 10-fold in OA or injured joints (Jones et al., 2008; Sekiya et al., 2012). These cells have been shown to have similar phenotypes to SM-MSCs; exhibiting significantly more clonogenicity and a lower adipogenic capacity *in vitro* than matched BM-MSCs (Jones et al., 2008). Gene profiles also demonstrate that SF-MSCs are more similar to SM-MSCs than BM-MSCs (Morito et al., 2008). Additionally, SF-MSCs express a similar panel of MSC surface antigens to SM-MSCs (see Table 1). The source of SF-MSCs may originate from shedding of joint structures such as the synovium, IFP or articular cartilage, the frequency of which increases as disease progresses.

#### Periosteum

Periosteum-derived progenitor cells (PDPCs) have been shown to display multipotency at single cell level and meet the criteria for classification as MSCs (De Bari et al., 2006; Choi et al., 2008). Periosteum is a specialized connective tissue that forms a membrane covering all bone surfaces except for articular cartilage and sesamoid bones. It consists of a fibrous external sheet and inner cambium layer where progenitor cells reside (Squier et al., 1990; Ferretti et al., 2012). To isolate stem cells, periosteum is surgically removed from bone and the cells liberated either enzymatically or through egression from small tissue explants. PDPCs have been identified using the classical MSC markers or clonally isolated using limiting dilution (Lim et al., 2005; De Bari et al., 2006; Choi et al., 2008). A very high percentage (>95%) of cells isolated from periosteum exhibit MSC marker expression (Lim et al., 2005; Chang and Knothe Tate, 2012). Periosteum-derived cell preparations can form cartilage, bone and muscle *in vitro* and *in vivo* (Gruber et al., 2001; De Bari et al., 2001b, 2006), as well as adipocytes *in vitro* (Sakaguchi et al., 2005; De Bari et al., 2006).

PDPCs can remain undifferentiated through many passages without losing their differentiation capacity (Ball et al., 2011). Periosteum-derived cells proliferate faster than most other MSCs *in vitro* (Chang and Knothe Tate, 2012; Radtke et al., 2013), linear growth kinetics are maintained for more than 30 population doublings that is concurrent with telomere length maintenance. PDPC do not show signs of senescence until 80 population doublings (De Bari et al., 2001b; Choi et al., 2008). Periosteum cells from aging patients have been shown to retain this high growth rate and differentiation capability unlike BM-MSCs that show decreased longevity, telomere shortening and senescence in aging donors (De Bari et al., 2001b, 2006; Stenderup et al., 2003; Lim et al., 2005; Stolzing et al., 2008).

BM-MSCs and PDPCs have different osteogenic, adipogenic, and chondrogenic potential. However, studies have reported contradictory data indicating both that PDPC are superior and inferior to BM-MSCs in their differentiation capacity (Yoshimura et al., 2007; Chang and Knothe Tate, 2012). These latter studies used different isolation protocols for PDPCs indicating the importance of standardized methodologies that allow cross-study comparison. The anatomical location of the periosteum plays an important role in its properties and thus the harvest site of PDPCs is important (Chang and Knothe Tate, 2012). An *in vitro* study of the periosteum's chondrogenic potential showed that PDPCs from the ilium, scapula, and tibia are capable of chondrogenesis, whereas periosteum from the skull exhibited no signs of chondrogenesis (Gallay et al., 1994). The variations seen in the differentiation capacities of PDPCs may be partly attributable to the differences in the periosteum developmental history.

Based on its accessibility during orthopedic surgery, and on the ability of periosteal cells to proliferate at much higher rates than other MSC sources, the periosteum is also an appealing cell source for tissue engineering approaches.

#### Infrapatellar fat pad

The IFP is a tubular piece of adipose tissue located below and behind the patella within the knee. Recent studies have demonstrated that the IFP is a source of adipose-derived MSCs and that these cells can be utilized for cell-based tissue engineering and treatment for cartilage regeneration (Dragoo et al., 2003; Wickham et al., 2003). It has been suggested that these cells have superior chondrogenic ability compared to MSCs derived from other tissues such as subcutaneous fat and bone marrow (Hindle et al., 2016). IFP-derived stem cells were shown histologically to produce more matrix than BM-MSCs and to have increased COL2A1, ACAN, and SOX9 gene expression (Hindle et al., 2016). Further information on adipose-derived MSCs is provided in the adipose tissue section.

#### Trabecular bone

Multiple groups have described a MSC population within trabecular bone with the characteristics of self-renewal and multilineage differentiation potential (Nöth et al., 2002; Sottile et al., 2002; Tuli et al., 2003a,b; Sakaguchi et al., 2004). MSCs were isolated from trabecular bone by digestion with collagenase and hence referred to as collagenase-released (CR) cells. The remaining bone fragments were then cultured and MSCs isolated as out-growing cells once 70–80% confluence was achieved. Stem cells isolated from trabecular bone have been shown to be virtually identical to MSCs obtained from marrow aspirates (Sakaguchi et al., 2004). Sakaguchi and colleagues found there were no obvious differences in MSC yield between young and elderly donors unlike BM-MSCs (Sakaguchi et al., 2004), though, their proliferation rate was found to decrease with age. Although trabecular bone-derived MSCs are capable of chondrogenic differentiation, they have not been widely used in regenerative strategies; this may be due to their elevated osteogenic potential or due to the invasive and protracted isolation methodologies.

#### Muscle

Skeletal muscle is known to contain progenitor cells, lineage restricted satellite cells, which are responsible for muscle regeneration. More recently, adult MSCs that are distinct from satellite cells and possess the ability to differentiate into other cell lineages have been discovered (Qu-Petersen et al., 2002; Usas and Huard, 2007; Zheng et al., 2007). Muscle-derived stem cells possess a high myogenic capacity and have been shown to regenerate both skeletal and cardiac muscle (Usas and Huard, 2007). *In vitro* these cells have been shown to be capable of differentiating into the osteogenic, chondrogenic, adipogenic, myogenic and endothelial lineages, and are capable of long-term proliferation and self-renewal (Jackson et al., 2010). Muscle-derived stem cells can be isolated via muscle biopsy from healthy tissue, surgical waste tissue from orthopedic reconstructions, or surgically debrided muscle tissue following orthopedic trauma (Qu-Petersen et al., 2002; Usas and Huard, 2007; Zheng et al., 2007; Nesti et al., 2008).

Several cell populations with the properties of MSCs have been identified in skeletal muscle, the best-characterized populations are muscle-derived stem cells (MDSCs) harvested from murine skeletal muscle. MDSCs are isolated using a pre-plating technique, an enrichment technique that eliminates more adherent cell types (Qu-Petersen et al., 2002), leaving cells capable of multilineage differentiation and self-renewal. This population has not been harvested from human muscle tissue using differential adhesion; instead, cells with similar *in vitro* characteristics have been isolated using positive expression for endothelial cell markers CD34 and CD144, and satellite cell marker CD56 (Zheng et al., 2007). This sub-population of satellite cells has been termed myoendothelial cells and are capable of osteogenic, adipogenic, and chondrogenic differentiation. Another distinct population of multipotent cells has been harvested from skeletal muscle following traumatic injury (Nesti et al., 2008). Unlike MDSC, these cells are rapidly adhering and present in high numbers during wound healing and remodeling; it is hypothesized that multipotent cells are recruited from their niche and proliferate to accomplish repair (Jackson et al., 2010). The cells harvested from the injured muscle are referred to as mesenchymal progenitor cells (MPCs) to indicate they may not have been in a quiescent, stem cell state when harvested (Jackson et al., 2010). MPCs have been shown to have very similar *in vitro* characteristics to MDSC and like bone marrow-derived MSCs can modulate the inflammatory response, promote angiogenesis and inhibit apoptosis of cells in close proximity *in vitro* (Djouad et al., 2010; Jackson et al., 2010). The final stem cell population within muscle are pericytes. Pericytes are associated with capillaries and microvessels in almost every tissue of the body. Isolated pericytes from muscle can differentiate into myocytes, osteoblasts, adipocytes and chondrocytes *in vitro* (Jackson et al., 2010) and repair muscle *in vivo*. It has been suggested that MSCs harvested from various tissues are in fact pericytes which originated in the vasculature of those tissues (Caplan, 2008).

Skeletal muscle is one of the most plentiful tissues in the body and given that these cells can be obtained with minimally invasive-biopsy procedures, there is growing evidence that skeletal muscle may be an important clinical source of MSCs for use in therapeutic applications (Usas and Huard, 2007). MDSCs, pericytes and MPCs have chondrogenic potential and might be suitable for cartilage regeneration. Muscle-derived MSCs have been shown to express SOX9, aggrecan, and type II collagen when differentiated into chondrocytes and when cultured at high density form defined pellets containing cells embedded in an ECM rich in sulphated proteoglycans and type II collagen (Farrington-Rock et al., 2004; Matsumoto et al., 2008). MDSCs transfected with BMP-4 have been shown to adopt a chondrogenic phenotype *in vitro* and when implanted into a full thickness osteochondral defects in rat knees resulted in persistent repair after 24 weeks (Kuroda et al., 2006). Additionally, MDSCs have been seeded and cultured in collagen type I scaffolds and implanted into osteochondral defects of New Zealand white rabbits knees (Adachi et al., 2002). That study found that after 24 weeks MDSCs gave improved regeneration of the articular surface, collagen type II expression and construct integration, compared with constructs seeded with chondrocytes (Adachi et al., 2002). These experiments demonstrate the potential of muscle-derived stem and progenitor cells for cartilage tissue engineering.

### Wharton's Jelly

MSCs isolated from within the Wharton's Jelly (hWJSCs) appear to have important advantages for regenerative medicine applications. These MSCs are relatively young given their isolation from discarded umbilical cord collected at birth, they can be harvested painlessly with no donor site morbidity, and they also co-express MSC and embryonic stem cell markers and therefore possess greater stemness properties. Wharton Jelly-derived MSCs have short population doubling time, exhibit non-lineage-restricted multipotency, are hypoimmunogenic and, most importantly, they do not form tumors in immunodeficient mice (Fong et al., 2010; Gauthaman et al., 2012). Independent research groups have successfully differentiated hWJSCs into chondrogenic lineage using conventional chondrogenic factors (Hoynowski et al., 2007; Wang et al., 2011; Reppel et al., 2015). Therefore, given the numerous advantages and the differentiation potential of hWJSCs, these could be considered as a potential alternative source for cartilage tissue engineering. For a comprehensive review of Wharton's Jelly-derived MSCs and their clinical applications see Kim et al. (2013).

## Chondrogenesis

Articular cartilage originally develops from multipotent progenitor cells in the presumptive skeletal embryonic mesenchyme. Under the influence of external and intrinsic factors, chondroprogenitor MSCs transform into chondroblasts and start depositing cartilage-specific ECM. Chondrogenesis of chondroprogenitor mesenchymal cells starts with the condensation and nodule formation and is highly dependent on the interplay between differentiating chondrocytes and their ECM. Understanding the mechanisms underlying the molecular control of chondrogenesis has important implications for the development of novel, effective treatment approaches and improving cartilage tissue engineering.

### Intracellular signaling pathways regulating chondrogenesis

Skeletal progenitor cells express the master chondrogenic transcription factor *SOX9*, which is required for cell survival, and maintenance of the expression of cartilage-specific markers (e.g., collagen types II, IX, and XI, as well as CD-RAP), enzymes involved in ECM production (e.g., chondroitin 4-sulfotransferase *CHST11*), its own partners in transcription (such as SOX5 and SOX6), as well as key signaling pathway mediators (e.g., fibroblast growth factor receptor-3, FGFR3) (Lefebvre and Dvir-Ginzberg, 2016). L-Sox5 and Sox6, two additional Sox transcription factor family members which contain no transcriptional activation domain, act in cooperation with SOX9 and are required for the expression of Col9a1, aggrecan, and link protein (Lefebvre et al., 2001). Given its quintessential activities in differentiating and mature chondrocytes, it is not surprising that *SOX9* is at the heart of many of regulatory pathways during chondrogenesis, including the hedgehog, FGF, TGF-β, BMP, Notch, and Wnt signaling pathways (Kozhemyakina et al., 2015). The *SOX9* promoter also contain binding sites for the hypoxia-inducible factor-1α (HIF1α), nuclear factor kappa B member RELA, and notch signaling mediator RBPj (Lefebvre and Dvir-Ginzberg, 2016). Sirtuin-1 (SIRT1), a NAD^+^-dependent deacetylase, is a positive regulator for chondrogenic differentiation of MSCs (Buhrmann et al., 2014). The Sox proteins are not the sole transcription factors that are active in chondroprogenitor cells; several other transcription factors such as Runt-related transcription factor-2 (Runx2) Barx2, Nkx3.2/Bapx1, Msx1 and 2, β-catenin, Smads, Lef1, AP-1, and AP-2 are also known to control chondrogenic differentiation (Goldring et al., 2006; Bobick and Kulyk, 2008; Bobick et al., 2009).

As a consequence of altered gene expression patterns following condensation, the ECM surrounding the differentiating chondroprogenitor cells also undergoes profound changes; cartilage-specific matrix components, most importantly collagen type II and aggrecan are deposited as chondrogenesis proceeds (Dessau et al., 1980). It is noteworthy that there is a reciprocal relationship between chondrocytes and their ECM, the characteristic composition and organization of the matrix is essential for maintaining the appropriate morphology, phenotype and function of differentiating and mature chondrocytes (Cancedda et al., 2000).

Alterations in the extracellular milieu mediated by the ECM are relayed to the intracellular signal transduction routes by reversible pathways. Transient protein phosphorylation is the most common posttranslational protein modification that has an influence on the activity of many signaling proteins. Among the intracellular factors that regulate chondrogenesis various Ser/Thr protein kinases and Ser/Thr phosphoprotein phosphatases were identified. All major Ser/Thr protein kinase family members including protein kinase A (PKA) (Yoon et al., 2000) and PKC (Matta and Mobasheri, 2014), as well as phosphoprotein phosphatases such as PP1, PP2A, and calcineurin (Matta et al., 2014) are essential regulators of chondrogenesis, with either stimulatory or inhibitory effects. Most importantly, mitogen-activated protein kinases (MAPKs) are key components of chondrocyte signaling involved in translating extracellular stimuli into cellular responses and coordinate proliferation, differentiation and gene expression. The role of MAPKs in chondrogenesis is the subject of several in-depth review articles (Bobick and Kulyk, 2008) and is only briefly discussed here. The three MAPK pathways contribute to the molecular control of chondrogenesis to a different extent; whilst JNKs seem not to be involved in this process, p38 and ERK1/2 are key regulators of chondrogenic differentiation. The p38 pathway is primarily involved in the initiation of condensation, whereas ERK1/2 interacts with BMP-2-induced signaling to promote chondrogenesis (Bobick and Kulyk, 2008). As far as the upstream mediators of the MAPK pathways are concerned, members of the TGF-β and FGF families, retinoic acid (RA), and integrins are reported to differentially activate p38 and ERK1/2 MAPK pathways (Stanton et al., 2003). In BM-MSCs, all three MAPKs were found to be positive mediators of TGF-β1-induced chondrogenesis by inducing N-cadherin expression (Bobick and Kulyk, 2008). The downstream targets of MAPK signaling include lineage-specific transcription factors including AP-1, ETS, Runx2, HIF-2α, and C/EBPβ (Lafont et al., 2007).

#### Chondrogenic differentiation of MSCs

**Figure 4:**
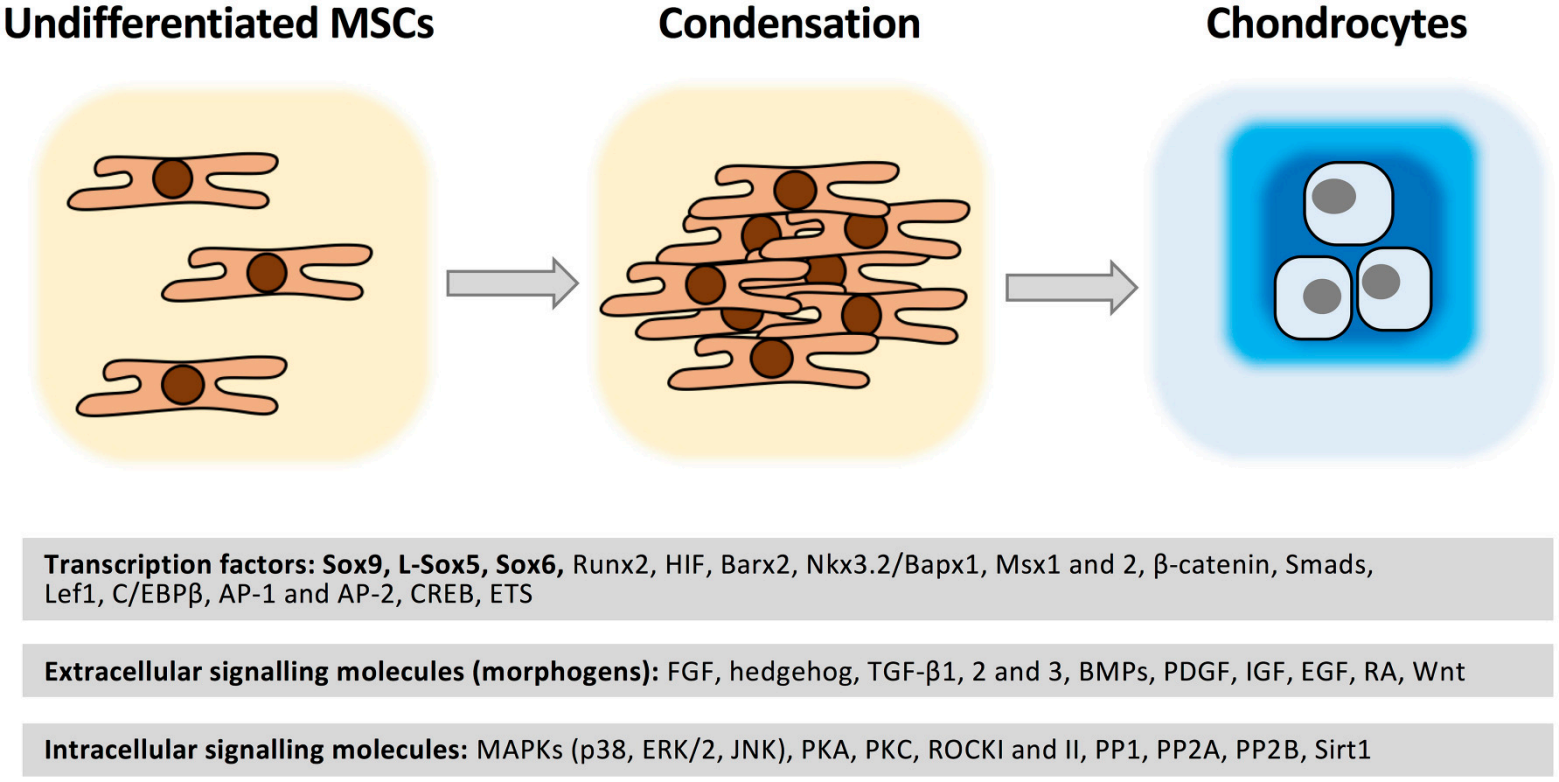
**Molecular regulation of chondrogenesis**. MSCs are recruited to the future sites of cartilage formation. Following migration and local proliferation cell density increases (condensation). Cell-cell contacts trigger a set of intracellular signaling events which result in chondrogenesis accompanied by a change in cell morphology and cartilage ECM molecule secretion. A wide range of transcription factors regulated by soluble extracellular signaling molecules acting through the modulation of various protein kinases/phosphoprotein phosphatases play essential roles in the molecular control of chondrogenesis. See further details in text. Please note that this list is not exhaustive.

*In vitro* chondrogenic differentiation of MSCs requires a combination of a 3D environment and external growth factor addition (Figure 4). Growth factors FGF, hedgehog, TGF-β, BMPs, platelet-derived growth factor (PDGF), insulin-like growth factor (IGF), epidermal growth factor (EGF), RA and the wingless/int (Wnt) glycoproteins have been used in various combinations to facilitate and enhance the efficacy of *in vitro* chondrogenic differentiation of MSCs. The glucocorticoid dexamethasone is used to further enhance cartilage-specific gene expression in TGF-β1 and -3 induced MSC cultures (Csaki et al., 2007; Bobick et al., 2009). Members of the TGF-β superfamily are potent inducers of chondrogenesis; TGF-β2 and TGF-β3 are more effective mediators than TGF-β1 (Archer et al., 2003b). BMP signaling is also necessary for chondrogenic differentiation; BMP-2 has the strongest stimulatory effect on chondrogenesis (Bobick et al., 2009); BMP-7 stimulates ECM synthesis and inhibits catabolic factors (Tuan et al., 2013). IGF-1 stimulates chondrogenic signal transduction by acting on collagen-binding β1-integrin-receptors and IGF-1-receptors activating the Ras–MAPK signaling pathway, and it has also been shown to stabilize the chondrogenic phenotype by stimulating SOX9 and promote molecular associations between ERK1/2 and SOX9 (Shakibaei et al., 2006).

MSCs cultured in chondrogenic conditions *in vitro* maintain their inherent developmental programme and undergo hypertrophy, which results in collagen types I and X secretion, in addition to gradual downregulation of *Col2a1* in the later stages of differentiation (Hardingham et al., 2006). In an attempt to avoid this, strategies based on the use of molecules with an inhibitory effect on growth plate development have being developed. For instance, the parathyroid hormone related peptide (PTHrP) or FGF-2 is known to downregulate *Col10a1*, but also *Col2a1*, during *in vitro* chondrogenesis of adult MSCs (Kim et al., 2008). The main challenge, therefore, is to prevent the formation of fibrous cartilage by inhibiting collagen type I expression and secretion, but at the same time maintain or increase collagen type II deposition during cartilage tissue regeneration techniques. In order to avoid fibrous cartilage formation during *in vitro* chondrogenesis, a more complete understanding of the intracellular signaling events is necessary.

## Perspectives and conclusions

Although initially considered as a tissue with a simple structure, reproducing the stratified appearance of cartilage has proven to be difficult. The task can be subdivided into two main phases, regeneration of cartilage tissue, achieved through mimicking embryonic chondrogenesis, and then remodeling of this repair tissue to generate the adult form and hence function. In an analogy to skin wound healing, which forms the longest part of the repair process, remodeling of the repair cartilage is predicted to take months or years, but, only through reconstitution of the original form can we expect to attain long-lasting and functional repair of osteochondral defects. We know that joint formation and maturation are driven by progenitor cells that exhibit the characteristics of MSCs, and therefore, these represent the best means to enable us to direct extrinsic repair processes through surgical intervention.

Historically, bone marrow-derived MSCs have been used to heal cartilage defects through implantation, however there are several important reasons why they are not optimal for durable repair strategies. First, BM-MSCs induce fibrocartilagenous repair, defined as matrix production that has a higher ratio of collagen type I to II than native tissue, and secondly, there is no evidence that this cartilage can be remodeled to form stratified hyaline cartilage. The presence of type I collagen is *per se* not deleterious, it is in fact normal for immature cartilage to be rich in type I cartilage in the superficial zone. Type I collagen expression is lost as the tissue matures and the protein cannot be immunolocalized in adult articular cartilage. Collagen type I and associated collagens types III and V reappear in injury and disease. The inherent problem with BM-MSCs is that collagen type I expression is an inherent part of their phenotype and transcriptional profile, hence the formation of fibrocartilagenous repair tissue. The most logical use of BM-MSCs in cartilage repair strategies would be in their native context, as a mesenchymal stromal cell, supporting repair of the local parenchymal cells, chondrocytes, through the supply of paracrine factors and generation of a provisional matrix. Similarly, there is scant evidence that other non-articular derived MSCs are capable of generating cartilage that can be remodeled to form stratified tissue, though each are capable of chondrogenic differentiation, and, producing paracrine factors that enable endogenous repair, particularly in fibrocartilagenous tissues such as menisci.

Thus far, articular cartilage-derived CPCs appear to have the attributes to produce repair tissue conducive to long-term repair. CPCs not only produce hyaline cartilage but also do not undergo terminal differentiation to produce calcifying cartilage. Most significantly, these cells are critical determinants in the maturational transition of cartilage from one that has an immature isotropic structure to one that is anisotropic and mature. In general, it has been predicted that maturation is essentially complete by puberty, which in rabbits is 3 months, and consequently much longer in humans. MRI imaging of ACI treated patients predicts that the remodeling phase is complete in 2 years, which gives a lower time estimate, although biopsies of these repair tissues indicate that the morphology of normal adult cartilage is seldom achieved. Work from a number of groups has shown that osteoarthritic cartilage contains a viable pool of CPCs that display all the qualitative attributes of MSCs. The question arises why do CPCs persist in diseased cartilage, and, can they be reactivated? We do not know whether the chondrogenic activity of CPCs from diseased cartilage is impaired *in vitro* compared to progenitors from normal cartilage. Similarly we are in the dark to whether epigenetic changes in OA CPCs resulting as a consequence of metabolic disease, chronic overloading, or genetic deficits affect their function. If this latter is shown to be the case, various reprogramming strategies to alter the epigenetic profile of these cells may induce reactivation of their chondrogenic phenotype. There is data to suggest that CPCs reside in a stem cell niche which is demarcated by unusual chondroitin sulfate epitopes. The loss of this niche through proteoglycan depletion following injury and disease may be a factor as to why CPCs become dysregulated and unable to differentiate, though data indicates self-renewal is not affected. Again reactivation of CPCs will have to take into account reestablishment of the niche, either through genetic reprogramming or presentation of the niche in biomaterials.

The presence of viable CPCs in diseased cartilage suggests that attempts to induce repair through endogenous mechanisms may be still within reach. MSCs from different origins may be useful in combination therapies where CPCs produce a hyaline repair tissue and adipose or bone marrow-derived MSCs provide supportive paracrine factors that enhance cell viability and reduce inflammation. When selecting the appropriate MSC source the balance will be between expediency and effectiveness. If we look at the options then almost every cell type has its' own advantages; ease of isolation, expansion, immunomodulatory effects, and chondrogenic capacity. In addition there are many challenges associated with isolating, expanding and differentiating MSCs for subsequent implantation into degenerate joints. The physiological microenvironment of diseased joints is hypoxic, acidic, deprived of nutrients, and contains high concentrations of pro-inflammatory cytokines and reactive oxygen species. Furthermore, MSCs may be exposed to abnormal physical loads as the joint has already been biomechanically compromised. Thus, future regenerative medicine strategies will need to address these remaining concerns possibly through addition of surgical interventions such joint realignment. As next steps, focusing efforts toward achieving standardized methods of MSC isolation, characterization, and administration has great potential to provide powerful new treatments. In addition, bioprinting technology offers the possibilities of delivering cells, growth factors, and biomaterials precisely to the required position and may be key to engineer hyaline cartilage. Given that patients present with different problems it is unlikely that there will be a simple “one size fits all” answer but probably an array of solutions that need to be carefully aligned to the individual's requirements.

## Author contributions

CF drafted the article. CM, RZ, IK, and AM contributed to the interpretation and critical appraisal of the literature, contributed text, and edited the manuscript. CM and CF prepared the original artwork. All authors have made substantial intellectual contributions to the evolution of the concepts presented in the manuscript and approved the final version submitted.

## Funding

The authors' work is supported by the European Commission and Arthritis Research UK. AM is the co-ordinator of the D-BOARD Consortium funded by European Commission Framework 7 programme (EU FP7; HEALTH.2012.2.4.5-2, project number 305815, Novel Diagnostics and Biomarkers for Early Identification of Chronic Inflammatory Joint Diseases). CM is supported by the New National Excellence Program of the Ministry of Human Capacities in Hungary, and the European Commission through a Marie Curie Intra-European Fellowship for Career Development grant (project number 625746; acronym: CHONDRION; FP7-PEOPLE-2013-IEF) awarded to AM. AM is also a member of the Applied Public-Private Research enabling OsteoArthritis Clinical Headway (APPROACH) Consortium, a 5-year project funded by the European Commission's Innovative Medicines Initiative (IMI). APPROACH is a public-private partnership directed toward osteoarthritis biomarker development through the establishment of a heavily phenotyped and comprehensively analyzed longitudinal cohort. The research leading to these results has received partial support from the Innovative Medicines Initiative (IMI) Joint Undertaking under grant agreement no. 115770, resources of which are composed of financial contribution from the European Union's Seventh Framework programme (FP7/2007-2013) and EFPIA companies' in kind contribution. AM has received funding from Arthritis Research UK (grant number 21076) and financial support from the Deanship of Scientific Research (DSR), King Abdulaziz University (grant no. 1-141/1434 HiCi). He is also member of the Arthritis Research UK Centre for Sport, Exercise, and Osteoarthritis, funded by Arthritis Research UK (Grant Reference Number: 20194). IK is supported by the UK Regenerative Medicine Platform.

### Conflict of interest statement

The authors declare that the research was conducted in the absence of any commercial or financial relationships that could be construed as a potential conflict of interest. The reviewer VT and handling Editor declared their shared affiliation, and the handling Editor states that the process nevertheless met the standards of a fair and objective review.
